# Posterior Hypopharyngeal/Upper Esophageal Wall Reconstruction Using a Double-Island Free Fasciocutaneous Anterolateral Thigh Flap: A Case Report and Scoping Review of the Literature

**DOI:** 10.3390/jcm14051779

**Published:** 2025-03-06

**Authors:** Léna G. Dietrich, Vera A. Paulus, Mihai A. Constantinescu, Moritz C. Deml, Roland Giger, Ioana Lese

**Affiliations:** 1 Department of Plastic and Hand Surgery, Inselspital, Bern University Hospital, University of Bern, 3010 Bern, Switzerlandmihai.constantinescu@insel.ch (M.A.C.); ioana.lese@insel.ch (I.L.); 2 Department of Orthopedics and Traumatology, Inselspital, Bern University Hospital, University of Bern, 3010 Bern, Switzerland; moritz.deml@insel.ch; 3 Department of Otorhinolaryngology, Head and Neck Surgery, Inselspital, Bern University Hospital, University of Bern, 3010 Bern, Switzerland; roland.giger@insel.ch

**Keywords:** posterior hypopharyngeal/upper esophageal wall defect, hypopharyngeal/upper esophageal reconstruction, double-paddle flap monitoring

## Abstract

**Background/Objectives**: Isolated defects of the posterior hypopharyngeal/upper esophageal wall are rare, typically arising after cancer resection or complications following cervical spine osteosynthesis. Various local and free flaps are available for reconstruction, but we opted for a double-island anterolateral thigh (ALT) flap in this case. **Methods**: A scoping review was conducted (June 2024) following PRISMAScR 2018 guidelines in order to examine the coverage options available in the literature for posterior hypopharyngeal/upper esophagus wall defects while also presenting a case where such a defect was covered with a double-island anterolateral thigh (ALT) flap. Eligibility criteria: Human studies describing defect coverage of the posterior hypopharyngeal/upper esophagus wall were included. Sources of evidence: A literature search was conducted in PubMed, Cochrane Library, and Google Scholar, following PRISMAScR guidelines. Charting methods: Data on surgical techniques, outcomes, and complications were extracted and analyzed by two independent reviewers. Case report: A 57-year-old female developed a chronic posterior wall perforation following Zenker’s diverticulum treatment and C5/6 cage osteosynthesis. Reconstruction was performed using a free fasciocutaneous ALT flap with two skin paddles: one (2 × 2 cm) for the esophageal mucosa and an additional vascularized fascia layer (4 × 8 cm) to separate the cage from the hypopharyngeal defect. To enable flap monitoring in the otherwise hidden defect, a second skin island was externalized cervically. **Results**: Postoperative recovery was uneventful, with a continuous viable flap signal. A Gastrografin swallow test confirmed an intact esophagus without leaks or dehiscences. Oral intake resumed after 10 days. The literature review highlighted 239 cases with multiple reconstructive techniques, each with advantages and limitations. **Conclusions**: The double-paddle free fasciocutaneous ALT flap is a viable option for posterior hypopharyngeal/upper esophageal wall reconstruction, allowing effective postoperative monitoring. This approach offers a valuable modification for complex cases requiring enhanced structural integrity and flap assessment.

## 1. Introduction

The reconstruction of the posterior hypopharyngeal and upper esophageal wall is a complex surgical challenge arising after oncologic resection or complications following cervical spine procedures. Isolated defects in this region are relatively rare but can result in significant morbidity due to impaired swallowing and increased risk of fistula formation or infections, particularly when osteosynthesis material is involved [1,2].

Hypopharyngeal and upper esophageal malignancies are among the more aggressive tumors in the head and neck region, frequently diagnosed at advanced stages due to their asymptomatic progression in the early phases [3]. Squamous cell carcinoma is the predominant histologic subtype, accounting for more than 95% of cases, with risk factors including tobacco use, alcohol consumption, and human papillomavirus (HPV) infection [4]. Posterior hypopharyngeal wall tumors specifically present unique challenges in resection and reconstruction due to their proximity to critical structures and rich lymphatic drainage, which predisposes them to early metastases [5,6].

Beyond oncologic cases, defects in the posterior hypopharynx and upper esophagus can also arise from complications of Zenker’s diverticulum surgery, cervical spine instrumentation, or radiation-induced necrosis [7,8]. Postoperative dehiscence in these cases can lead to severe complications, including mediastinitis and chronic aspiration. Therefore, reconstruction strategies must aim not only to restore anatomical integrity but also to ensure functional outcomes such as swallowing and phonation while minimizing donor site morbidity [9].

Several reconstructive techniques have been described, ranging from primary closure and pedicled flaps to free tissue transfer. Local options such as the pectoralis major myocutaneous flap or supraclavicular artery island flap provide robust coverage but may be inadequate for posterior defects or cases involving hardware exposure, especially in already irradiated or operated cases [10,11]. Free flaps, particularly the radial forearm free flap (RFFF) and anterolateral thigh (ALT) flap, have been increasingly favored for their thin, pliable tissue and reliable vascularization, making them suitable for complex reconstructions [12,13]. In addition to anatomic reconstructive approaches, it is important to mention the possibility of a diversion loop, which provides an alternative pathway for food continuity. One example is the free jejunal flap, particularly in patients with impaired swallowing and aspiration [14]. However, in our case, we focused on the anatomic reconstruction of the posterior hypopharyngeal/upper esophageal wall and, therefore, chose not to further elaborate on this option.

We were faced with the challenge of reconstructing a posterior hypopharyngeal/upper esophageal wall defect ensuing after the resection of a Zenker’s diverticulum via an open approach in the presence of an intervertebral titanium cage and anterior plate at the C5/6 cervical spine level. Since the region underwent multiple orthopedic operations, a local flap was not an option anymore. Therefore, a free flap with feasible postoperative monitoring options was needed. This led to the idea of using two skin paddles: one to cover the hypopharyngeal/upper esophageal defect and the second to serve as a monitor island at the neck level.

The purpose of this study is to present a posterior hypopharyngeal/upper esophagus wall defect coverage by means of a free double-paddled anterolateral thigh (ALT) flap, showing the feasibility of this technique for delicate reconstructions requiring pliable tissues and the possibility of monitoring at a hidden localization. This method is a modification of the well-established ALT flap technique. Additionally, a scoping literature review was also conducted according to the PRISMAScR 2018-guidelines in order to examine and identify the coverage options available in the literature for posterior hypopharyngeal/upper esophageal wall defects in the context of trauma, the presence of osteosynthesis, or other etiologies, as no single standardized approach has been established.

## 2. Case Report

We present the case of a 57-year-old female patient with chronic dysphagia due to a Zenker’s diverticulum (Brombart grade 3), with a history of a failed attempt at a rigid and flexible transoral cricopharyngeal myotomy and subsequent surgical treatment involving a left transcervical approach, dissection, and excision of the Zenker’s diverticulum along with cricopharyngeal myotomy in March 2023. Additionally, she had a history of cervical spondylosis and foraminal stenosis at C5/6, treated with anterior cervical fusion and cage placement in 2018, followed by cage removal and revision in 2019 due to a pseudarthrosis. A new cage and anterior plate were installed at the C5/6 level. In January 2024, the patient was diagnosed with a chronic hypopharyngeal/upper esophageal wall perforation and dehiscence with exposure of the cervical spine’s anterior plate and suspected chronic instability and infection of the cervical vertebrae underlying the current cage.

Her medical history was complex and extended, marked by numerous complications stemming from both the initial surgical interventions and subsequent attempts at management. These challenges necessitated a highly specialized multidisciplinary approach to achieve adequate tissue coverage while ensuring the highest level of safety and durability in such a delicate and anatomically concealed area. The reconstruction of a transmural defect of the posterior hypopharyngeal/upper esophageal wall requires thin, well-vascularized, and stable tissue, with donor sites offering low morbidity, ensuring safety for a high-risk patient with a history of multiple prior surgeries. Given the unique location of the defect—on the posterior wall of the hypopharynx/upper esophagus, an area that is difficult to access and visualize—the surgical team faced a critical question: could the reconstruction be successfully achieved using a free flap, specifically one that could be visually monitored for viability postoperatively?

Despite the absence of clear guidelines or case reports addressing this specific situation in the literature, we chose to proceed with an innovative surgical strategy. The solution involved the use of a free ALT flap, fashioned with two separate skin paddles. One paddle was to be employed for the reconstruction of the hypopharyngeal/upper esophageal posterior wall defect. The second paddle was supposed to be positioned externally, serving as a “monitor paddle”, allowing for real-time visual assessment of the flap’s viability during the critical postoperative period. The technical execution of this procedure required meticulous planning, given the dual objectives of reconstructing the hypopharyngeal/upper esophageal wall while simultaneously providing sufficient coverage for the osteosynthesis material at the C5/6 level.

**Figure 1:**
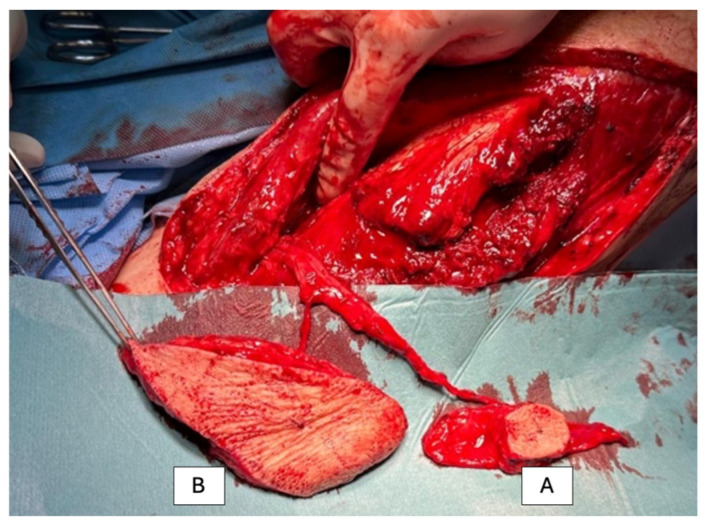
Harvesting of the free ALT flap from the right thigh. Before clamping the pedicle, the flap is prepared for the subsequent reconstruction. Two skin paddles are created for coverage: Skin paddle A (2 × 2 cm skin, 4 × 8 cm fascial tissue) is used as reconstructive tissue and sutured into the posterior wall of the esophagus, with the fascial tissue providing sufficient size to cover the osteosynthesis material. Skin paddle B is designated as a monitor and will be sutured between the skin wound edges on the left neck side.

**Figure 2:**
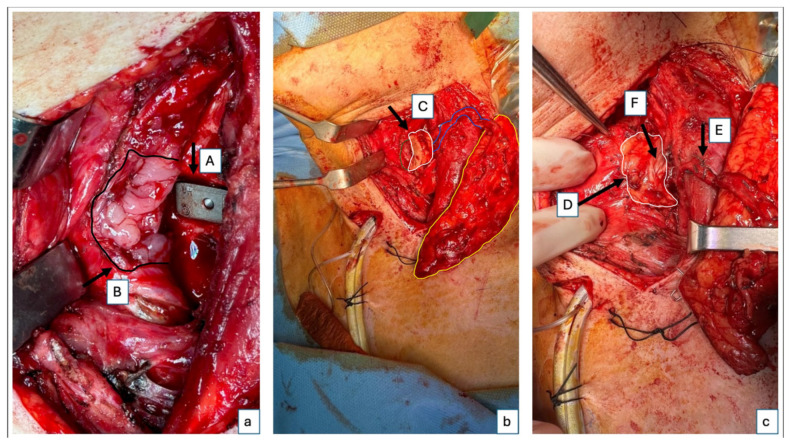
Intraoperative images of flap inset. Intraoperative images after suturing of the smaller skin paddle for reconstruction of the posterior hypopharyngeal/upper esophageal wall. The second, larger skin paddle has not yet been sutured and will be positioned cervically as a monitoring paddle. (**a**) A close-up of the cage (A) and the posterior hypopharyngeal/upper esophageal defect marked with the black arrow and delineated by the black line (B). (**b**) Right lateral fixation of the flap to the defect boarder; the left lateral border of the defect is delineated with the green line, while the small skin paddle used for the defect reconstruction, the monitoring skin paddle, and the perforator going from the monitoring paddle to the skin paddle used for the posterior hypopharyngeal/upper esophageal wall reconstruction are delineated by a white, yellow, and blue line, respectively (C). (**c**) Complete fixation of the flap in the defect, also on the left side, with a second layer of suture for the fat tissue—the white line delineates the skin paddle used to completely close the posterior hypopharyngeal/upper esophageal wall defect (D); perforator location of the small skin island (E); additional vascularized fascia for cage coverage (F).

In February 2024, an interdisciplinary surgical intervention was performed following a multidisciplinary board consensus in this complex case involving cervical implant instability, a chronic implant-associated infection of the cervical osteosynthesis material, a chronic hypopharyngeal/upper esophageal fistula, and a hypopharyngeal/upper esophageal defect requiring reconstruction. The procedure began with the Otolaryngology team performing a left cervicotomy and freshening the wound edges. Subsequently, the Orthopedics team revised the cervical spine, removing the existing plate and intervertebral cage, followed by the implantation of a new anterior cervical titanium 3D-printed highly porous cage at the C5/6 level. Intraoperative probes from the soft tissue around the spine and the bone of C5 and C6 vertebrae were collected for histologic and microbiologic analysis. The Plastic Surgery team harvested a 6 × 16 cm microvascular ALT flap from the right thigh. The two skin paddles were meticulously prepared, and vascularization of both skin paddles was checked at the donor site before clamping the pedicle (Figure 1). The first skin paddle, measuring 2 × 2 cm, was used to reconstruct the hypopharyngeal/upper esophageal mucosal defect, while a 4 × 8 cm section of vascularized fascia from the perforator was positioned between the posterior hypopharyngeal/upper esophageal wall and the cage to provide an additional protective layer (Figure 2). Despite the relatively small size of the mucosal defect, prior surgeries had left the tissue fibrotic and too rigid to be closed primarily without risking tension, dehiscence, or stenosis. This fascia placement also helped prevent future complications by acting as a buffer between the esophagus and the cervical spine hardware.

The second skin paddle, based on a separate perforator, was externalized to the neck region for postoperative monitoring. This was a critical decision, as the location of the posterior hypopharyngeal/upper esophageal wall made visual inspection impossible without an externalized monitor island. While some may argue that flow-through couplers could be used as a monitoring tool, the literature indicates that their reliability can be inconsistent. Relying solely on them in such a high-risk area would have posed significant risks to the patient’s recovery. Furthermore, local flap options like the platysma or supraclavicular flaps were not viable due to the patient’s history of multiple surgeries.

**Figure 3:**
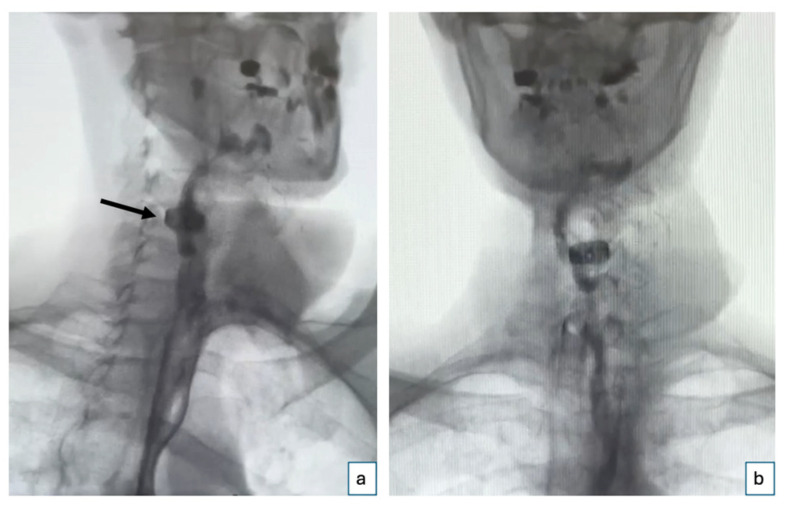
Gastrografin swallow test. Postoperative Gastrografin swallow test performed on the 9th postoperative day shows an intact esophagus with no evidence of leakage or wound dehiscence: (**a**) oblique right view (the black arrow indicates the position of the cage) and (**b**) anteroposterior view.

**Figure 4:**
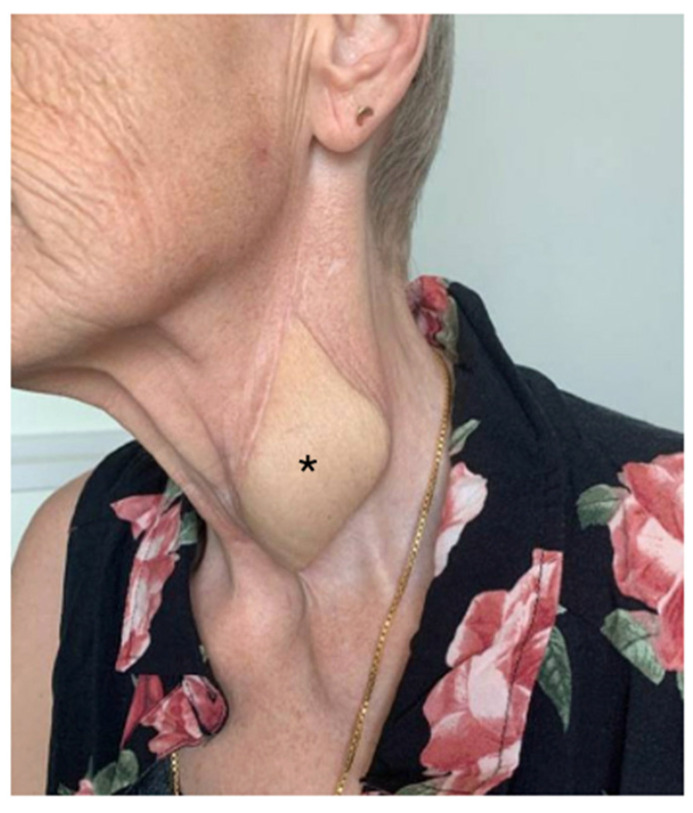
Postoperative follow-up. Four months postoperatively, with unremarkable scar conditions and excess skin; * marks the monitor spindle.

Postoperatively, the patient’s recovery was uneventful. The flap maintained strong signals throughout, and a Gastrografin swallow test performed 9 days after surgery could exclude any leakage and wound dehiscences (Figure 3). The patient started drinking and successively eating orally 10 days after the procedure. Four months postoperatively (Figure 4), the patient demonstrated a stable course with no complications at the recipient site. The patient reported no dysphagia.

## 3. Scoping Review of the Literature

### 3.1. Methods

**Figure 5:**
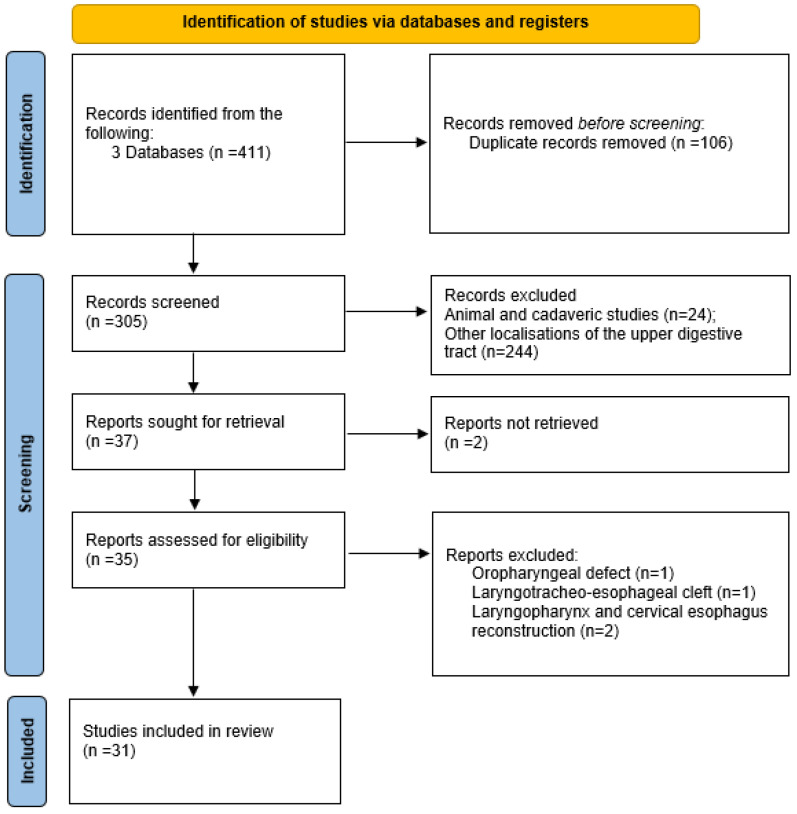
A PRISMAScR diagram of the scoping literature review.

The methodological framework for this scoping review was a literature search conducted on 20 June 2024, using PubMed, Cochrane Library, and Google Scholar, following PRISMAScR guidelines. The study selection process is summarized in a PRISMAScR flow diagram (Figure 5). The search strategy focused on identifying studies related to the reconstruction of the posterior esophageal or pharyngeal wall. It used a combination of keywords and MeSH terms, including “posterior”, “esophageal”, “pharyngeal”, and “reconstruction”, with Boolean operators to ensure comprehensive coverage of the relevant literature. To ensure reproducibility, the exact search query used in PubMed was as follows: “posterior” [All Fields] AND (“esophageal” [MeSH Terms] OR “pharyngeal” [MeSH Terms]) AND “reconstruction” [All Fields]. No filters or restrictions (e.g., publication date, study type, or full-text availability) were applied to allow for a comprehensive assessment of all the relevant literature. No restrictions were applied regarding publication years to ensure a comprehensive assessment of all the available literature. Two independent reviewers evaluated the identified articles by screening titles, abstracts, and full texts. Additional studies were included by reviewing the references of relevant articles. Any disagreements between the reviewers were resolved through discussion and consensus. The search strategy was specifically designed to include only primary research studies, excluding reviews, book chapters, and expert opinions. The following variables were extracted: (1) study design (retrospective/prospective), (2) patient population characteristics (age, comorbidities), (3) type of surgical technique used (local vs. free flaps, specific flap type), (4) reported surgical outcomes (functional swallowing, phonation, fistula formation, infection rates), and (5) complications (categorized as minor or major). Where specific outcome definitions varied across studies, data were standardized by categorizing complications into “minor” (requiring conservative management) and “major” (requiring reoperation). No assumptions were made regarding missing data, and only explicitly reported variables were included in the final analysis. Data from the included studies were extracted using a standardized data charting form, which included variables on study design, patient population, surgical techniques, outcomes, and complications. The form was developed based on key parameters identified in previous reviews and was tested for consistency before use. Data charting was performed independently by two reviewers, and discrepancies were resolved through discussion. No direct contact with study investigators was made for additional data confirmation. Articles published in English and German across any journal were considered, and data on surgical techniques, outcomes, and complications were collected. Animal studies, cadaveric studies, and other localizations of the upper digestive tract were excluded. An extensive cross-check of the references from the original publications was performed to identify additional articles. Due to the nature of this study, formal ethics application and approval by the institutional review board were not required. No prior review protocol was registered for this scoping review.

### 3.2. Results

**Figure 6:**
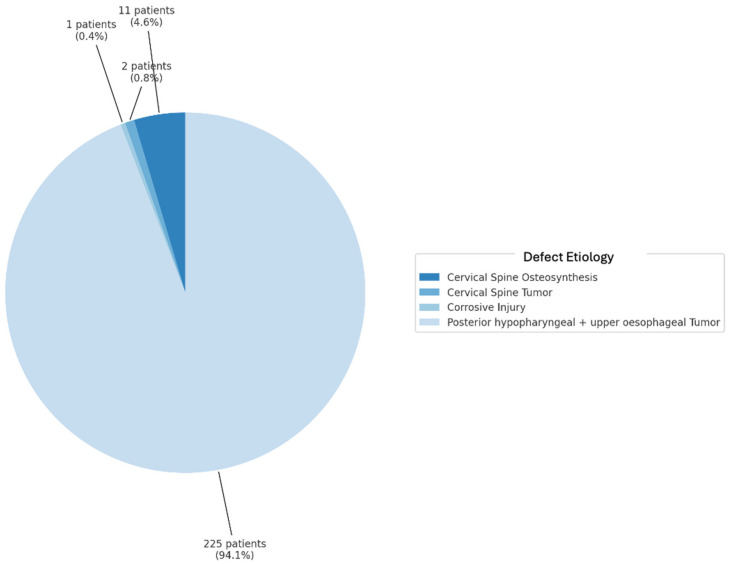
Defect etiology in percentages.

**Figure 7:**
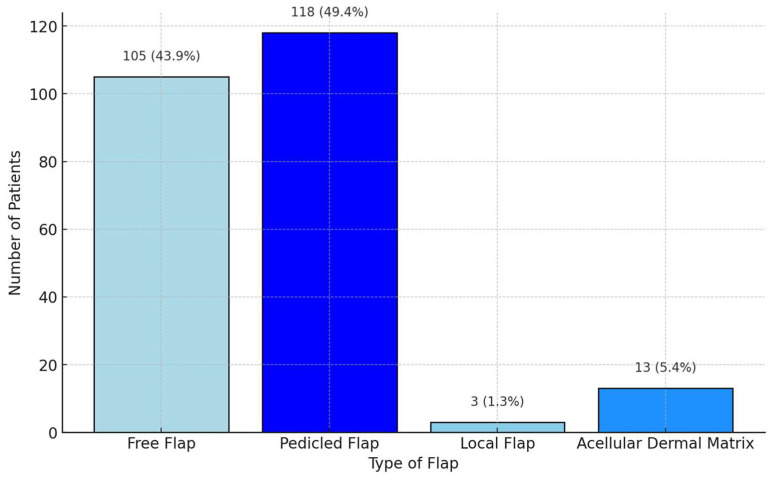
Grouped distribution of reconstruction methods.

**Figure 8:**
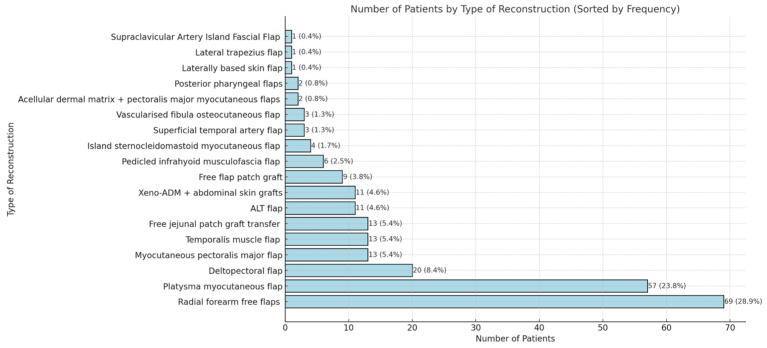
Detailed distribution of reconstruction methods. Xeno-ADM: Xeno-Acellular Dermal Matrix; ALT: adipofascial anterolateral thigh.

**Figure 9:**
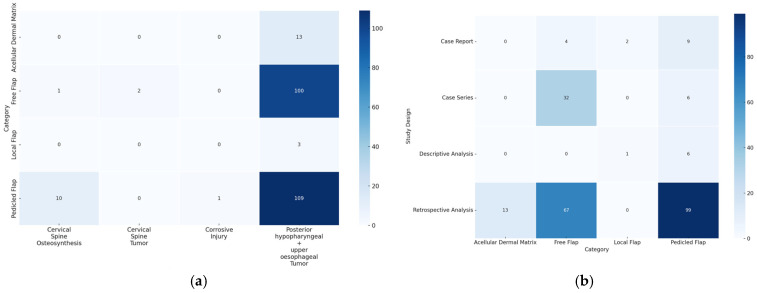
(**a**) Heatmap of categories by defect etiology. The y-axis displays the different reconstructive categories (local, pedicled, free flap, and acellular dermal matrix), while the x-axis represents the defect etiology. The color coding indicates the frequency: the darker the color, the more frequently the corresponding combination of category and defect etiology occurs. (**b**) Heatmap of study designs by categories. The y-axis displays the study designs, while the x-axis represents the different reconstructive categories (local, pedicled, free flap, and acellular dermal matrix). The color coding indicates the frequency: the darker the color, the more frequently the corresponding combination of study design and category occurs.

The scoping review revealed a variety of reconstructive options for posterior hypopharyngeal/upper esophageal wall defects, ranging from pedicled to free tissue transfers, each with their own advantages and limitations. In our scoping review, 31 of the initial 411 publications treating the reconstruction of posterior hypopharyngeal/upper esophageal defects were included in the final analysis with a total number of 239 patients in 31 publications (225 hypopharyngeal/upper esophageal cancer cases, 11 complications related to osteosynthesis of the spine, 2 spine tumor cases, 1 case after corrosive injury (Figure 6)). The study characteristics, including study design, patient population, surgical techniques, and outcomes, are detailed in Table 1. Posterior hypopharyngeal/upper esophageal wall reconstruction has been performed using various types of flaps (Figure 7 and Figure 8): 105 free flaps and 118 local/pedicled flaps with a mean follow-up time of 26 months (1.5–72 months). Furthermore, two autologous hypopharyngeal/upper esophageal patch techniques and two studies with multiple procedures or other materials (two) were included. Heatmaps of study designs by categories and types of reconstruction by defect etiology are provided in Figure 9. Extracted data were summarized using descriptive statistics, including frequencies for categorical variables (e.g., surgical techniques and complications). Data were organized into figures/tables and visualized through heatmaps to illustrate patterns in reconstructive approaches and complication rates. Complications and revisions related to the flaps are summarized in Table 2. In the thirteen patients treated with acellular dermal matrix, one pharyngeal fistula occurred, with no revisions reported. In total, the complication rate for the local, pedicled, and free flaps was 0% (0/3), 32.2% (38/118), and 19% (20/105), respectively. However, despite the complications described, not every paper reported the revisions performed and what exactly was conducted every time, accounting for the limitations of the retrospective studies.

## 4. Discussion

This study revealed different etiologies for posterior wall defects of the hypopharyngeal/upper esophageal region (posterior hypopharyngeal/upper esophageal carcinoma, spine surgery, chordoma, corrosive trauma) as well as different reconstructive options. In contrast to the small number of cases described in the literature, a wide range of techniques are described. With twelve case reports, five case series, twelve retrospective studies, and two descriptive studies, there are various models that shed light on this topic. The use of a free double-paddle flap for a posterior hypopharyngeal/upper esophageal wall defect has been described only once in the literature [2]. The follow-up of our patient revealed minimal morbidity at the donor site, a patient-centered solution, and a satisfactory postoperative course, with no dysphagia reported 4 months postoperatively.

The reconstruction of the hypopharyngeal and hypopharyngeal/upper esophageal regions after oncologic resection presents significant challenges due to the need to restore critical functions such as swallowing, phonation, and airway integrity. This review explores a variety of reconstructive techniques, including local flaps, pedicled flaps, free flaps, and synthetic materials, each with distinct advantages and potential complications.

### 4.1. Local Flaps

Historically, in 1974, Seda described a surgical approach for carcinomas of the posterior hypopharyngeal/upper esophageal and hypopharyngeal wall, where reconstruction was achieved with a laterally based skin flap in two stages. His technique preserved a functioning larynx and reestablished the continuity of the upper alimentary tract. This historical approach marked the beginning of using vascularized flaps for reconstructing the posterior hypopharyngeal/upper esophageal wall [3]. The posterior pharyngeal flap has proven to be a reliable and effective method for reconstructing cervical hypopharyngeal/upper esophageal defects, enabling the restoration of hypopharyngeal/upper esophageal continuity in a single-stage operation, with minimal postoperative complications, such as fistula formation [4,5]. The local flap option is reserved for smaller defects and is not suitable for covering osteosynthesis material, as required in our patient’s case.

### 4.2. Pedicled Flaps

Myocutaneous flaps, such as the pectoralis major flap, have been successfully used for posterior tracheal wall reconstruction, offering stability and reliable coverage in complex cases with extensive resections [6,27]. This flap remains a well-established option for large, circumferential defects, particularly in patients who are poor candidates for microvascular surgery. It provides reliable bulk and coverage for large defects; however, its bulk can sometimes impair functional outcomes, particularly in terms of swallowing and speech recovery [7,25].

For cases requiring thinner, more pliable tissue, the supraclavicular artery island flap has gained prominence. Studies have shown that this flap offers reliable, thin tissue with minimal donor site morbidity, making it ideal for reconstructing moderate-sized defects with functional demands [10,29]. The platysma flap, while less commonly used, offers a simpler alternative for smaller defects, with good functional outcomes and minimal morbidity. It provides a one-stage solution for reconstructing smaller posterior hypopharyngeal/upper esophageal wall defects, offering minimal donor site morbidity and acceptable functional outcomes in swallowing and respiration [7,20,24,28]. A notable limitation of supraclavicular and platysma flaps is their dependence on a neck that has not been subjected to multiple prior surgeries.

The lateral trapezius flap presents another option for reconstructing extensive posterior hypopharyngeal/upper esophageal wall defects, with successful outcomes in maintaining airway patency and swallowing function in complex cases [15]. The temporalis muscle flap, with its robust blood supply and versatility, has also shown promise in reconstructing posterior hypopharyngeal/upper esophageal wall defects. It provides flexible coverage and reduces donor site morbidity compared to other regional flaps. While less commonly used, it has demonstrated reliable outcomes for restoring swallowing and airway patency without compromising speech [17]. Despite these advancements, complications such as fistula formation and flap necrosis remain significant challenges across all reconstructive methods [29]. While both of these options seem viable, head and neck reconstruction is nowadays more focused on the “reconstructive elevator”, with free tissue transfer being the first choice in such cases, in order to disturb the surrounding tissues as little as possible. Moreover, the lateral trapezius flap could be associated with accessory nerve problems, while donor site morbidity after temporalis muscle harvest entails contour deformity and an extensive undermined area in order to place the flap in the desired defect.

The deltopectoral flap continues to be a reliable and versatile option for head and neck reconstruction, particularly for posterior hypopharyngeal/upper esophageal wall defects, due to its technical simplicity and consistent survival rates [6]. However, the pedicle of the flap remains visible, therefore deforming the sternoclavicular area. Newer methods, such as the island sternocleidomastoid myocutaneous flap, have also shown promising results in managing posterior hypopharyngeal/upper esophageal wall defects after cervical spine surgeries, but they are indicated in cases without previous neck surgeries [25,34].

### 4.3. Free Flaps

Free flaps are a cornerstone in reconstructive surgery, particularly for addressing complex defects with extensive tissue loss and functional demands. Due to the necessity of two skin paddles, Fritz et al. reported their experiences, like our case, with a free double-paddle posterior tibial artery flap. The indication was a leakage of the hypopharynx after bougienage, which occurred through stenosis due to radiotherapy after tumor resection. Compared to our case, where no external skin was required, soft tissue with a skin paddle was also required in the neck area, as previous operations and radiotherapy had led to severe fibrosis of the neck. A purposeful result was achieved, and the authors appreciated the possibility of monitoring the otherwise hidden flap reconstruction [2]. This work differs from our technique in that the monitoring skin paddle was intentionally used to provide additional soft tissue coverage for the neck, whereas in our case, this was not necessary, and we plan to remove the skin paddle 1–2 years postoperatively. Moreover, we consider the thickness of a lower-leg free flap a disadvantage for posterior hypopharyngeal/upper esophageal wall reconstruction, where thin tissue is necessary.

The radial forearm free flap (RFFF) remains a gold standard for larger and more complex reconstructions due to its pliability, long vascular pedicle, adaptability to different anatomical regions, and suitability for irradiated areas [20]. It is particularly useful for posterior hypopharyngeal/upper esophageal wall defects and has been associated with faster recovery of swallowing and decannulation [9,18,20,22]. Reconstruction with RFFF in posterior hypopharyngeal/upper esophageal wall resections demonstrated functional voice preservation and acceptable morbidity in healthy patients, though patients with significant comorbidities exhibited increased complications [21]. However, the donor site morbidity of the RFFF is often criticized. Basaran et al. published original work in 2020 about the reconstruction of posterior esophagus defects using radial forearm flaps. Compared to our result (10 days after surgery), the patients achieved oral intake in a median time of 74 days after surgery. The authors concluded that tumor resection does not compromise the swallowing function permanently if hypopharyngeal/upper esophageal wall reconstruction is performed with a free flap [16]. RFFF reconstruction was also shown to achieve acceptable functional results in patients undergoing larynx-preserving posterior pharyngectomy, with six out of seven patients resuming oral intake, although long-term PEG dependence remained a risk for patients with swallowing difficulties [19]. The U-shaped RFFF has been identified as a reliable technique also for reconstructing the hypopharynx, offering functional benefits such as a well-maintained swallowing process in most patients while minimizing complications associated with jejunal grafts [11].

The adipofascial anterolateral thigh (ALT) flap is another option that offers thinner, more pliable tissue than the pectoralis major muscle flap (PMMF) and avoids bulk, which can compromise functional recovery [12,26]. It is particularly beneficial for smaller, complex defects requiring both functional and aesthetic considerations [26]. The ALT flap is highlighted as an effective option for complex reconstructions, providing adequate tissue volume and a robust vascular supply, particularly beneficial in challenging anatomical regions [30]. However, the skin can be reliably preserved in order to provide an even more stable inner lining of the esophagus. Fasciocutaneous free flaps, such as the ALT flap or RFFF, have been effectively utilized for hypopharyngeal/upper esophageal wall reconstruction, with favorable oncologic outcomes and an 84% success rate in restoring oral nutritional intake [8]. One-stage reconstruction utilizing a free vascularized fibula steocutaneous flap has also been described for simultaneous restoration of the posterior hypopharyngeal/upper esophageal wall and cervical spine, offering a comprehensive solution for complex defects [23]. However, the fibula flap was not considered necessary in our case as no bone reconstruction was required, and the patient’s slim physique made the ALT flap an excellent option due to its versatility and adequacy for soft tissue reconstruction.

Free jejunal patch graft transfer after partial hypopharyngectomy has demonstrated satisfactory swallowing function with low complication rates, making it a viable reconstruction option for posterior hypopharyngeal/upper esophageal wall defects. Notably, 95% of patients resumed oral intake, with outcomes varying slightly based on defect extent [13]. Similarly, the free jejunal graft is an established technique for circumferential hypopharyngeal/upper esophageal defects, providing early restoration of oral intake and low rates of fistula formation [22,33]. However, the intraabdominal access can increase morbidity postoperatively.

### 4.4. Acellular Dermal Matrix

The use of a xenogeneic acellular dermal matrix (xeno-ADM) in combination with traditional flaps, such as the PMMF, offers an alternative technique that reduces donor site morbidity and promotes faster recovery of swallowing function [32,33]. ADM is suitable when well-vascularized tissue is available as a foundation; however, in our case, the presence of an osteosynthesis plate rendered this option inapplicable.

### 4.5. Venous Flow Coupler in Head and Neck

We are aware that medical technological innovations are available for monitoring buried flaps and that these techniques are also utilized in the head and neck region [35]. The study by Troob et al. highlights the reliability and utility of the venous flow coupler in monitoring free flap reconstructions in the head and neck region. With a sensitivity of 90% and specificity of 94.9%, the device proved highly effective, particularly with its negative predictive value of 98.9%, ensuring the reliable identification of viable flaps. Among 217 cases, 20 required revisions for flap compromise, with 16 successfully salvaged. Notably, the coupler detected five compromised flaps before arterial signal changes occurred, enabling timely interventions and improved salvage rates. Although the false-positive rate was 5.1%, it increased to 24.1% when two couplers were used simultaneously, likely due to complex flow dynamics. The coupler’s ability to continuously monitor venous flow makes it particularly valuable for detecting venous thrombosis early, even in the absence of arterial signal loss. Despite occasional challenges, such as false signals due to backflow, the venous flow coupler remains a reliable tool for enhancing the early detection of complications and improving flap salvage outcomes [35]. Nevertheless, a residual risk of limited reliability remains. In this patient with multiple previous operations, we specifically aimed to minimize the risk of false-negative monitoring and the associated need for unnecessary revision surgery.

Various techniques for posterior hypopharyngeal/upper esophageal wall reconstruction, including free flaps like radial forearm, fibula, and ALT flaps, have been described, each with specific advantages and disadvantages. An individualized multimodal approach tailored to each patient is crucial, and the choice depends on defect size, comorbidities, and prior radiotherapy [31]. Our described technique, while innovative and effective, requires specialized microvascular expertise, limiting its use in centers without advanced reconstructive capabilities. Long-term functional outcomes, such as swallowing and phonation, warrant further investigation. Future studies should evaluate flap durability, especially in high-risk patients [34].

### 4.6. Limitations and Future Directions

The main criticism of this technique is the actual reliability of the monitoring skin island, since it can also provide false-negative or false-positive signals, potentially misleading the assessment of the reconstructive component.

Further, this study is limited by the rarity of isolated posterior hypopharyngeal/upper esophageal wall defects, restricting generalizability. Long-term outcomes, including stricture formation and functional recovery, require further study. Additionally, the scoping review process was limited by the variability in study designs, differences in reporting standards, and potential publication bias, which may have influenced the availability of comparable data. The exclusion of non-English and non-German studies may have further impacted the comprehensiveness of the findings. Future research should focus on improving other advanced monitoring methods since multicenter validation and statistically relevant comparative studies are rather impossible due to the rarity of and the variability in these types of defects.

## 5. Conclusions

Several methods have been proposed for reconstructing the posterior hypopharyngeal/upper esophageal wall, though no single technique has demonstrated clear superiority. Our case report suggests that posterior hypopharyngeal/upper esophageal wall reconstruction with a double-island free fasciocutaneous ALT flap is feasible and allows monitoring in the postoperative course in an otherwise not visible area, when one of the islands is buried (for reconstructive purposes) and the other one is externalized (for monitoring purposes). Our approach offers a potential solution for cases involving complex hypopharyngeal/upper esophageal defects and osteosynthesis material coverage, particularly when traditional methods are unsuitable. Future studies should evaluate long-term functional outcomes, complication rates, and comparative efficacy with other reconstructive techniques in larger patient cohorts.

## Figures and Tables

**Table 1:** Overview of studies on posterior hypopharyngeal/upper esophageal wall reconstruction.

Study	Year	Study Design	Patient Population	Surgical Techniques	Outcomes
Agarwal et al. [15]	2016	Case report	Synovial sarcoma	LTMF	Successful reconstruction; mild aspiration; disease-free survival
Alvarez et al. [7]	1997	Retrospective analysis	Advanced head and neck tumors	Platysma flap	36% survival; 9% necrosis; 27% complications
Basaran et al. [16]	2020	Retrospective analysis	Hypopharyngeal carcinoma; larynx preservation surgery	RFFF	Successful oral intake median 74 days; decannulation median 90 days
Bootz et al. [11]	2002	Retrospective analysis	Hypopharyngeal and laryngeal malignancies	RFFF	18 normal swallowing; n = 6 esophageal speeches; n = 1 flap necrosis; 2 stenosis (total 20)
Chan et al. [6]	2014	Retrospective analysis	Recurrent pharyngeal carcinoma patients	DPF	Improved functional outcomes; 96.3% flap survival
Coelho et al. [12]	2017	Case report	C5–C6 fracture and anterior cervical spine fusion	ALT	Successful reconstruction: normal swallowing restored; no complications at 3-year follow-up
Gibson et al. [10]	2020	Case report	Esophageal perforation post ACDF	SCAIF	Flap repair; initial recovery and consecutive complications
Hanasono et al. [17]	2001	Retrospective analysis	Head and neck oncologic defects	TMF	No flap loss; mild donor site morbidity; low complication rates; some cases of partial dehiscence and transient velopharyngeal insufficiency
Iyer et al. [18]	2009	Case report	Recurrent skull base chordoma	Fibula	Simultaneous bone stability and pharyngeal integrity
Jol et al. [19]	2003	Case series	Advanced posterior pharyngeal wall carcinoma	RFFF	All maintained voice function; 6/7 resumed oral intake
Joo et al. [8]	2012	Case series	Hypopharyngeal squamous cell carcinoma	RFFF and ALT	Disease-specific survival of 67%; recurrence in 15 patients (35%); n = 2 patients successfully salvaged; functional outcomes showed 84% oral alimentation and 88% decannulation rate
Julieron et al. [20]	2001	Retrospective analysis	Squamous cell carcinoma of the posterior pharyngeal wall	Multiple (thoracic myocutaneous, platysma, RFFF)	5-year survival 35% (primary surgery + radiotherapy); 16% (salvage surgery); 96% decannulated; 89% resumed oral intake; high complication rate in previously irradiated cases
Li et al. [4]	2019	Case report	Hypopharyngeal carcinoma	PPF	Successful reconstruction; no necrosis; good phonation, swallowing, and respiratory function.
Lydiatt et al. [21]	1996	Case series	Posterior pharyngeal wall carcinoma	RFFF	High complication rate (6/9); major complications in ASA 3 patients; n = 3 patients achieved full oral nutrition; n = 4 were decannulated; all retained voice function
Miyamoto et al. [13]	2011	Retrospective analysis	Primary and recurrent hypopharyngeal cancers	FJPG	95% resumed oral intake; n = 2 required tube feeding; 15% complication rate; all decannulated; 40% survived without recurrence
Nakatsuka et al. [22]	1997	Retrospective analysis	Carcinoma of the posterior wall of the hypopharynx	FJPG and RFFF	89% laryngeal preservation; n = 1 flap necrosis; n = 6 resumed oral intakes ≤ 3 weeks; n = 3 disease-free ≥ 5 years; n = 3 died
Ng et al. [23]	2002	Case report	Squamous cell carcinoma	Fibula	Restoring cervical spine stability and pharyngeal and laryngeal integrity and function
Suárez Nieto et al. [24]	1983	Descriptive analysis	Advanced head and neck tumors	Platysma flap	Successful reconstruction; minor complications
Pfitzmann et al. [25]	2003	Case report	Corrosive injury	PMF	Successful reconstruction
Rodriguez-Lorenzo et al. [26]	2013	Case report	Cervical spine and pharyngeal defect	Fibula	Restored deglutition
Rubinstein et al. [3]	1975	Case report	Congenital upper esophageal stenosis	PPF	Successful reconstruction; normal liquid diet post operation
Salmerón-González et al. [27]	2018	Case report	Squamous cell carcinoma	DPF	Pharyngocutaneous fistula (day 13), resolved spontaneously (day 34)
Seda et al. [5]	1974	Descriptive analysis	Advanced head and neck tumor	Laterally based skin flap	Successful reconstruction; no major complications; good swallowing and voice function
Seidl et al. [28]	2006	Case report	Esophageal reconstruction cases	IMF	Functional recovery; varied success
Shen et al. [29]	2011	Case report	Hypopharyngeal carcinoma; larynx-preserving surgery	TMF	No flap loss; no fistula; mean decannulation at 6 months; speech function rated excellent
Soares et al. [30]	2010	Retrospective	Advanced carcinoma of the posterior pharyngeal wall	DPF	Oral swallowing within three–four months
Spiro et al. [31]	1990	Retrospective analysis	Advanced head and neck cancer	Multiple (free flap, pedicle flap)	32% 5-year survival; 50% complications
Steinhart et al. [9]	1998	Case series	Advanced carcinoma of the posterior hypopharyngeal wall	RFFF and FJPG	Larynx preserved in all; oral swallowing: 7/9 (≤3 months), 1/9 (4 months), 1/9 gastrostomy; decannulation: 6/9; resection: R0 (7), R1/R2 (2); recurrence: 3; deaths: 3
Zang et al. [32]	2020	Retrospective analysis	Posterior hypopharyngeal wall cancer	ADM and abdominal skin grafts	Fast eating function recovery
Zhang et al. [33]	2010	Retrospective analysis	Advanced hypopharyngeal carcinoma	ADM with PMF	No pharyngeal fistula; some pharyngeal stenosis, but all patients resumed normal diet
Zhang et al. [34]	2021	Retrospective analysis	Complex head and neck defects	SMF	Effective reconstruction; good postoperative function

PPF: posterior pharyngeal flap; SMF: sternocleidomastoideus myocutaneous flap; SCAIF: Supraclavicular Artery Island Fascial Flap; PMF: pectoralis major flap; TMF: temporalis muscle flap; DPF: deltopectoral flap; LTMF: lateral trapezius myocutaneous flap; IMF: infrahyoid muscle flap; RFFF: radial forearm free flap; ALT: adipofascial anterolateral thigh; FJPG: free jejunal patch graft; n: numerical indication

**Table 2:** Complications and revisions for local, pedicled, and free flaps (n = 226 *).

	Technique Used (n = 226)	Complications	Reported Revisions
**Local flap** **(n = 3)**	- PPF (n = 2) - Skin flap (n = 1)	None	None
**Pedicled flap** **(n = 118)**	- Platysma flap (n = 57) - DPF (n = 20) - TMF (n = 16) - PMF (n = 13) - IMF (n = 6) - SMF (n = 4) - SCAIF (n = 1) - LTMF (n = 1)	- Platysma flap (25/57) **: hematoma (1), cervical abscess (7), fistula (15), total flap loss (7), partial flap loss (5), necrosis and infection (8), and cervical dehiscence (3) - DPF (6/20): fistula (4), partial flap loss (tip) (1), and infection (1) - TMF (5/16): paralysis of the temporal branch of the facial nerve (1), dehiscence (2), and transient velopharyngeal insufficiency (2) - PMF (6/13) **: abscess (3), fistula (2), flap loss (2), and wound infection (2) - SCAIF (1/1): hematoma (1)	- DPF (2/20): infection (1) and pharyngeal fistula (1)
**Free flap** **(n = 105)**	- RFFF (n = 77) - FJPG (n = 22) - ALT (n = 3) - Fibula (n = 3)	- RFFF (17/77) **: postoperative death (1), Adult Respiratory Distress Syndrome (ARDS) (1), cardiac failure (1), transient hypoglossal nerve paralysis (1), fistula (2), flap loss (4), hematoma (4), abscess (6), and fistula (2) - FJPG (4/22): infection (2), fistula (1), and flap loss (1) - ALT (1/3): hematoma (2)	- RFFF (7/77): hematoma (4), stenosis (2), and flap loss (1) - ALT (1/3): hematoma (1)

PPF: posterior pharyngeal flap; SMF: sternocleidomastoideus myocutaneous flap; SCAIF: Supraclavicular Artery Island Fascial Flap; PMF: pectoralis major flap; TMF: temporalis muscle flap; DPF: deltopectoral flap; LTMF: lateral trapezius myocutaneous flap; IMF: infrahyoid muscle flap; RFFF: radial forearm free flap; ALT: adipofascial anterolateral thigh; FJPG: free jejunal patch graft. * Thirteen patients with ADM were excluded. ** Some patients with multiple complications were included.

## Data Availability

Not applicable.
